# Effects of traditional Chinese exercises on cardiac rehabilitation in patients with myocardial infarction: a meta-analysis of randomized controlled trials

**DOI:** 10.3389/fcvm.2023.1223677

**Published:** 2023-10-02

**Authors:** Jingfang Zhang, Jieqiong Weng, Mengfei Yuan, Xingchen Shen, Yayi Weng, Xiaoxu Shen

**Affiliations:** Department of Cardiology, Dongzhimen Hospital, Beijing University of Chinese Medicine, Beijing, China

**Keywords:** traditional Chinese exercise, tai chi, ba duan jin, cardiac rehabilitation, myocardial infarction, meta-analysis

## Abstract

**Objective:**

Traditional Chinese exercises (TCE) are excellent cardiac rehabilitation (CR) training that can effectively improve cardiorespiratory fitness. However, there is no published meta-analysis of TCE on CR in patients with myocardial infarction (MI). Therefore, this study aimed to provide a comprehensive evaluation from multiple perspectives.

**Methods:**

This meta-analysis is based on the Cochrane Handbook of Systematic Reviews. Eight databases were searched from the date of database construction to March 15, 2023. Two investigators independently screened the literature and assessed their quality. The meta-analysis was performed with RevMan5.4 software.

**Results:**

A total of 21 articles involving 1,890 patients were included. N-terminal pro-brain natriuretic peptide (NT-proBNP) in the TCE group were lower than the control group (MD = −96.34, 95%CI: −140.69 ∼−51.98, *P* < 0.00001, *I*^2 ^= 96%), the left ventricular ejection fraction (LVEF) in the TCE group was higher than the control group (MD = 4.58, 95%CI: 3.28–5.88, *P* < 0.00001, *I*^2 ^= 79%), the left ventricular end diastolic dimension (LVDD) in TCE group was lower than the control group (MD = −3.83, 95%CI: −5.27 ∼−2.38, *P* < 0.00001, *I*^2 ^= 94%), the left ventricular end systolic diameter (LVESD) in TCE group was lower than the control group (MD = −2.17, 95%CI: −4.10 ∼−0.24, *P* < 0.00001, *I*^2 ^= 96%), The 6-minute walk test (6MWT) in the TCE group was higher than the control group (MD = 69.60, 95%CI: 34.59–104.60, *P* < 0.00001, *I*^2 ^= 99%), the oxygen uptake (VO_2_) in the TCE group was higher than the control group (MD = 4.38, 95%CI: 2.25–6.51, *P* < 0.00001, *I*^2 ^= 94%), the 36-item short form survey (SF-36) in the TCE group was higher than the control group (MD = 13.34, 95%CI: 9.25–17.42, *P* = 0.008, *I*^2 ^= 75%), the Hamilton Anxiety Scale (HAMA) in the TCE group was lower than the control group (MD = −4.34, 95%CI: −5.18 ∼−3.50, *P* = 1.00, *I*^2 ^= 0%), the Hamilton Depression Scale (HAMD) in the TCE group was lower than the control group (MD = −3.48, 95%CI: −5.35 ∼−1.61, *P* = 0.0002, *I*^2 ^= 88%), the incidence of major adverse cardiac events (MACEs) in the TCE group was lower than the control group (RR = 0.31, 95%CI: 0.20–0.47, *P* = 0.52, *I*^2 ^= 0%). Subgroup analysis revealed differences in TCE types could be a potential source of heterogeneity.

**Conclusion:**

MI patients who used TCE have not only notable improvements in cardiopulmonary function, physical function, quality of life, and emotions but also reduced the incidence of MACEs. Tai Chi might be more efficient than Ba Duan Jin.

**Systematic Review Registration:**

https://www.crd.york.ac.uk/PROSPERO/, identifier CRD42023408675.

## 1. Introduction

Cardiovascular disease (CVD) accounts for a large proportion of a range of non-communicable diseases. Acute myocardial infarction (AMI), one of the primary causes of death from coronary heart disease (CHD) (1), affects approximately 11.39 million individuals in China, where the prevalence of CVD is continually rising (2). The usual treatment for AMI often involves percutaneous coronary intervention (PCI) and coronary artery bypass grafting (CABG), which can enhance the quality of life for myocardial infarction (MI) patients by restoring perfusion. However, how to recover the pre-morbid health status remains an urgent public health issue (2). Cardiac rehabilitation (CR) is a comprehensive multidisciplinary program specifically designed for patients with CVD. The main objectives are to increase daily function and lessen cardiovascular risk factors (3). The European Society of Cardiology, American Heart Association, and American College of Cardiology all suggest utilizing CR as a Class I standard of care following an AMI (4).

Traditional Chinese Exercise (TCE) is a type of therapeutic, aerobic, mind-body exercise that has a history dating back over 3,000 years from traditional Chinese medicine (5). TCE mainly includes Tai Chi, Ba Duan Jin, Qi Gong, Yi Jin Jing, Wu Qin Xi, and other low to medium-intensity exercises (6). As a mild muscle-strengthening sport, TCE combines spiritual meditation with moderate postures, musculoskeletal stretching, and deep breathing (7, 8). It has been shown in numerous studies to be an effective exercise for CR and to enhance cardiorespiratory health (9, 10).

Early and appropriate CR not only improves cardiac function and quality of life but also prognosis in MI patients. However, there is no published meta-analysis evaluating the effect of TCE in these patients. Accordingly, the purpose of this study is to systematically evaluate the effects from multiple perspectives and provide a resource for future clinical advice.

## 2. Materials and methods

The Cochrane Handbook of Systematic Reviews served as the foundation for our meta-analysis. The results of this study followed the Preferred Reporting Items for Systematic Reviews and Meta-Analyses (PRISMA) statement. This meta-analysis has been registered in PROSPERO (registration number: CRD42023408675).

### 2.1. Search strategy

The relevant literature published in Pubmed, Web of Science, Cochrane Library, Embase, China Knowledge Network (CNKI), Vipshop database (VIP), Wanfang Knowledge Service Platform, and Sinomed Database from inception to March 15, 2023, were searched. The following search terms were utilized: (((((((traditional Chinese exercise [Title/Abstract]) OR (tai chi [Title/Abstract])) OR (tai ji [Title/Abstract])) OR (ba duan jin [Title/Abstract])) OR (yi jin jing [Title/Abstract])) OR (wu qin xi [Title/Abstract])) OR (qi gong [Title/Abstract])) AND (myocardial infarction [Title/Abstract]) AND (randomized [Title/Abstract]).

### 2.2. Study eligibility

Inclusion criteria: (1) The type of study is a randomized controlled trial (RCT). (2) The subjects were patients with a definite diagnosis of MI. (3) The intervention in the treatment group was TCE, such as Tai Chi, Ba Duan Jin, Qi Gong, Wu Qin Xi, or Yi Jin Jing. (4) The control group received conventional treatment or other exercises. (5) The effectiveness outcomes included N-terminal pro-brain natriuretic peptide (NT-proBNP), left ventricular ejection fraction (LVEF), left ventricular end diastolic dimension (LVDD), left ventricular end systolic diameter (LVESD), 6-minute walk test (6MWT), oxygen uptake(VO_2_), the 36-item short form survey (SF-36), Hamilton Depression Scale (HAMD), Hamilton Anxiety Scale (HAMA) and major adverse cardiac events (MACEs).

Exclusion criteria: (1) Duplicate literature. (2) Animal experiments, meta-analyses, and systematic reviews. (3) Studies with insufficient data, such as those in which the data could not be combined with other outcome indicators.

### 2.3. Data extraction

Two investigators independently screened the retrieved literature. Firstly, eliminate duplicate publications. Then read titles and abstracts to exclude the literature not relevant to the topic. Finally read the full text to identify whether the literature met the inclusion criteria and establish the information base, including author, country, year of publication, general information, interventions, outcomes, and indicators for conducting the quality evaluation. Discrepancies between investigators were resolved by discussion or a third independent reviewer.

### 2.4. Quality assessment

Cochrane Handbook (11) was used to assess the quality of the included literature for random sequence generation, allocation concealment, blinding, incomplete outcome data, selective reporting, and other biases. Each entry was evaluated for one of low risk, high risk, and unclear risk.

### 2.5. Statistical analysis

The meta-analysis was performed with the RevMan5.4 software. Dichotomous variables were expressed as relative risk (RR) and 95% confidence interval (CI), and continuous variables were expressed as mean difference (MD) and 95% CI. *I*^2^ was used to determine the heterogeneity. Meta-analysis was conducted using the fixed-effects model if *P* > 0.1 and *I*^2 ^< 50%. Meta-analysis was conducted using the random-effects model if *P* ≤ 0.1 and *I*^2 ^≥ 50%. Sensitivity analysis and subgroup analysis were used to examine the source of heterogeneity. The publication bias was displayed in a funnel plot. The test level of the meta-analysis was set as *α* = 0.05.

## 3. Results

### 3.1. Search results and population characteristics

**Figure 1:**
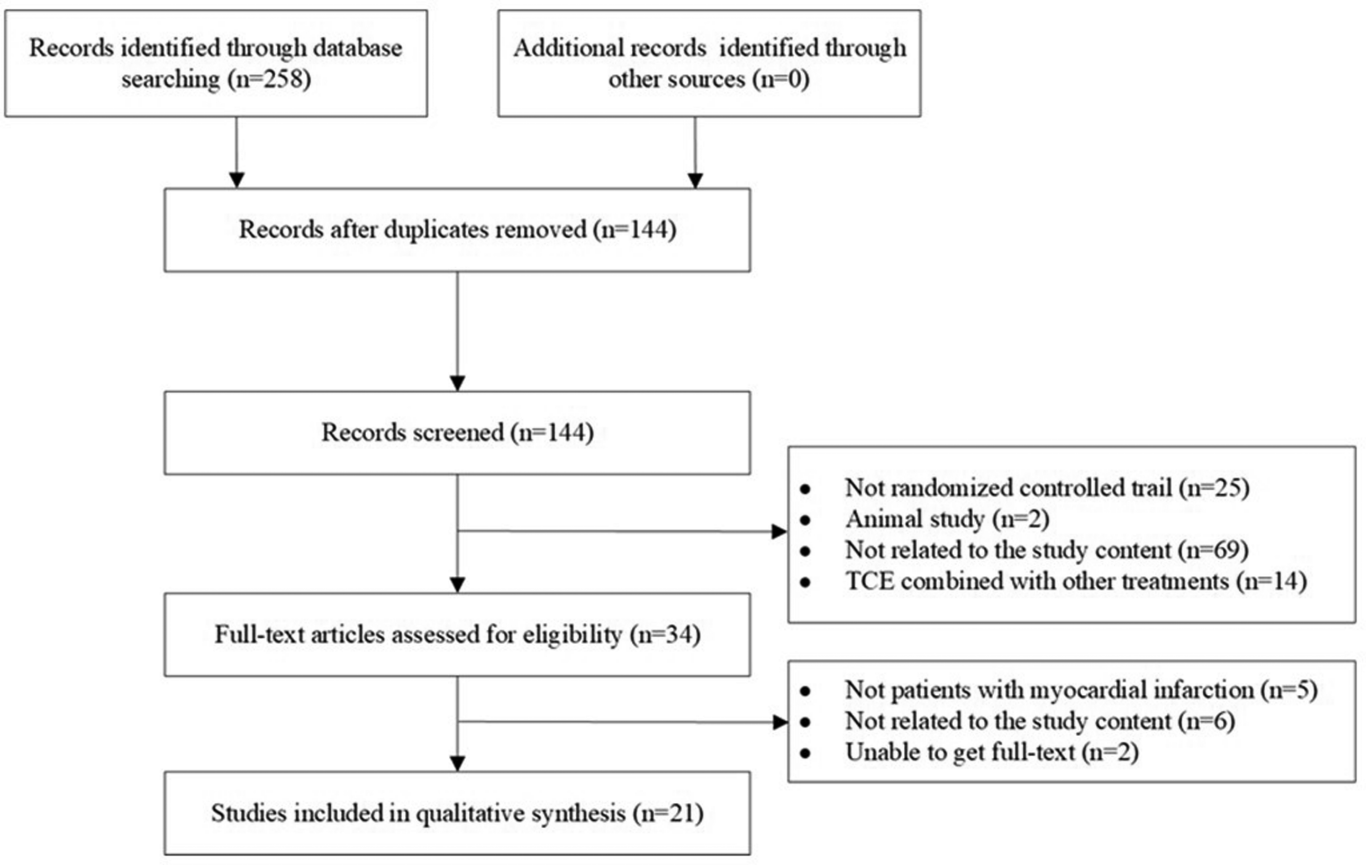
Flow chart of the search strategy.

The study screening process is illustrated in Figure 1. A total of 258 articles were searched through the databases (21 from Pubmed, 28 from Web of Science, 20 from Embase, 29 from Cochrane Library, 48 from CNKI, 33 from Wan Fang, 49 from VIP, and 30 from Sinomed). After removing duplicates and screening, 21 articles revolving 1,890 patients were eligible for meta-analysis. The general characteristics are summarized in Table 1. According to the table, 17 studies were from China and one was from Brazil. Interventions included Ba Duan Jin, Tai Chi Quan, and Tai Chi Ball. Intervention duration ranged from two weeks to one year.

**Table 1:** Characteristics of the clinical trials included in the meta-analysis.

Number	Study	Country	Patients	n (T/C)	Age (T/C, years old)	Intervention (T/C)	Duration	Outcomes
1	Wang et al. (12)	China	AMI	75/75	59.3 ± 15.4/58.8 ± 12.5	CT and Ba Duan Jin/CT and conventional exercise	6 months	①③④⑤⑩
2	Cai et al. (13)	China	AMI	45/45	49.58 ± 9.41/49.47 ± 9.32	CT and Ba Duan Jin/CT	2 weeks	⑤⑥
3	Wang et al. (14)	China	AMI	40/40	59.54 ± 14.78/58.65 ± 17.38	CT and Ba Duan Jin/CT	2 weeks	⑦
4	Zhang et al. (15)	China	AMI	35/35	59.42 ± 7.022/58.65 ± 7.027	CT and Ba Duan Jin/CT	8 weeks	①②
5	Zhou et al. (16)	China	AMI	50/50	57.87 ± 4.61/58.41 ± 4.52	CT and Ba Duan Jin/CT	3 months	①③④⑤
6	Zong et al. (17)	China	AMI	50/50	57.26 ± 6.84/56.91 ± 7.34	CT and Ba Duan Jin/CT	2 weeks	①③④⑥⑦⑩
7	Kang et al. (18)	China	AMI	30/30	51.27 ± 10.62/51.33 ± 9.95	CT and Ba Duan Jin/CT	2 months	①③⑤⑧⑨
8	Liu et al. (19)	China	STEMI	30/30	56.21 ± 10.44/57.32 ± 11.36	CT and Ba Duan Jin/CT	12 weeks	①②⑤
9	Guo and Jia (20)	China	AMI	60/60	58.21 ± 8.12/58.01 ± 9.54	CT and Ba Duan Jin + Tai Chi Ball/CT	1 month	①②⑨⑩
10	Li (21)	China	AMI	30/30	60.33 ± 12.34/60.13 ± 9.78	CT and Ba Duan Jin + Tai Chi Ball/CT	2 months	①②⑥⑧⑨
11	Yang (22)	China	AMI	69/69	63.19 ± 5.62/64.11 ± 5.64	CT and Tai Chi Ball/CT	4 weeks	⑥
12	Yu et al. (23)	China	AMI	32/32	65.36 ± 4.12/66.33 ± 5.35	CT and Tai Chi Quan/CT	4 months	①②③⑤
13	Zhang and Chan (24)	China	AMI	66/66	56.4 ± 4.3/55.7 ± 4.26	CT and Tai Chi Quan/CT	1 year	①⑤⑦
14	Wang et al. (25)	China	STEMI	30/30	55.25 ± 11.13/54.86 ± 12.05	CT and Tai Chi Quan/CT	6 months	②⑦
15	Liu et al. (26)	China	AMI	40/40	<60 years old, 60–70 years old, ≥70 years old: 18, 8, 14/16, 9, 15	CT and Tai Chi Quan/CT	12 weeks	①②⑩
16	Lu (27)	China	AMI	48/48	65.30 ± 2.45/66.07 ± 2.84	CT and Tai Chi Quan + Ba Duan Jin/CT	3 months	①③⑤⑩
17	Yu et al. (28)	China	AMI	53/53	61.13 ± 11.06/60.4 ± 11.37	CT and Ba Duan Jin/CT	6 months	①③④⑤
18	Li et al. (29)	China	AMI	29/28	58.52 ± 5.95/57.57 ± 6.69	CT and Tai Chi Quan/CT	6 months	⑤
19	Mao et al. (30)	China	AMI	56/54	60.43 ± 10.21/61.30 ± 11.12	CT and Ba Duan Jin/CT	12 weeks	①③
20	Chen et al. (31)	China	AMI	48/48	59.98 ± 10.86/61.49 ± 11.54	CT and Ba Duan Jin/CT	24 weeks	①
21	Rosane et al. (32)	Brazil	AMI	31/31	56 ± 9/60 ± 9	CT and Tai Chi Quan/CT + spinal stretching exercise	12 weeks	⑥

AMI, acute myocardial infarction; STEMI, ST-segment elevation myocardial infarction; T, traditional Chinese exercise rehabilitation group; C, control group; CT, conventional treatment; ① LVEF ② NT-proBNP ③ LVDD ④ LVESD ⑤ 6MWT ⑥ VO_2_ ⑦ SF-36 ⑧ HAMA ⑨ HAMD ⑩ MACEs.

### 3.2. Quality assessment

The results of the risk of bias evaluation of the included literature are presented in Supplementary Table S1. Two studies (12, 28) employed random samplings, 16 studies utilized random number table grouping and three studies (24–26) did not describe the randomization method. Only Rosane (32) used blinding of outcome assessment.

### 3.3. Meta analysis

#### 3.3.1. Effects of TCE on Nt-proBNP

**Figure 2:**
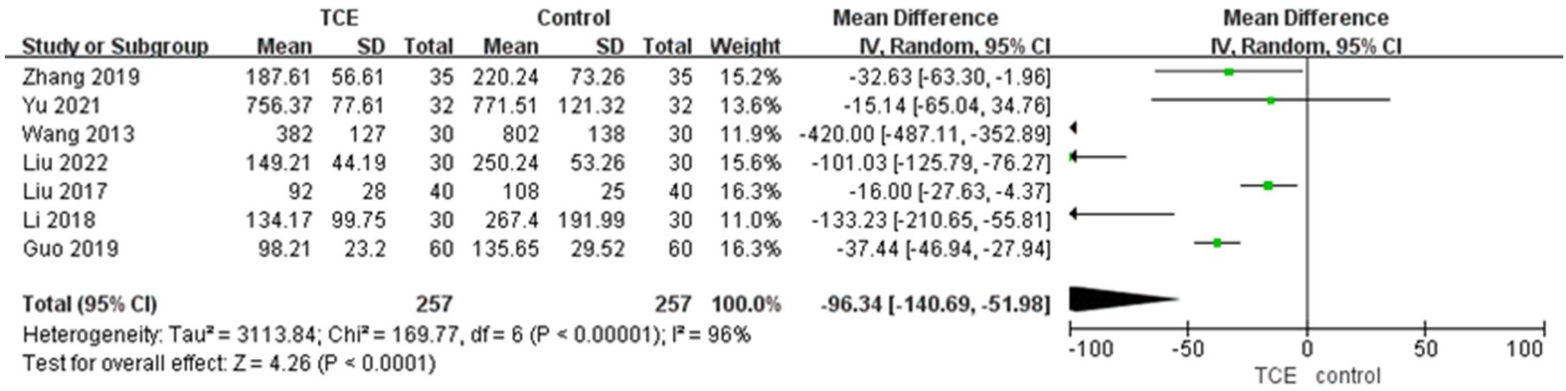
The forest plot of NT-proBNP.

Seven articles involving 514 patients reported NT-proBNP levels. The NT-proBNP of patients who engaged in TCE was significantly less compared with the control group based on a random-effects model (MD = −96.34, 95%CI: −140.69–−51.98, *P* < 0.00001, *I*^2 ^= 96%) (Figure 2).

#### 3.3.2. Effects of TCE on echocardiographic indicators of cardiac function

**Figure 3:**
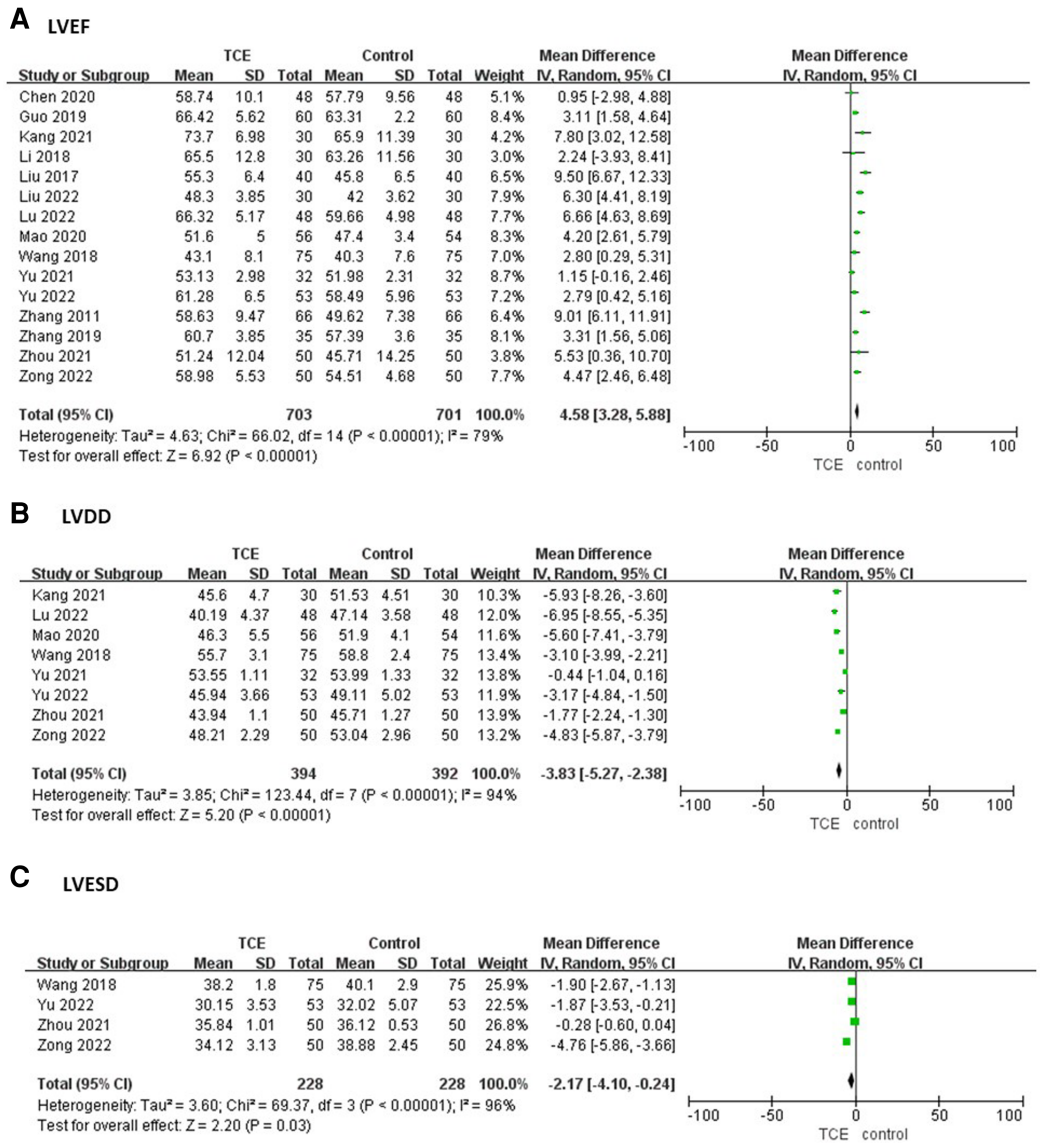
The forest plots of (**A**) LVEF, (**B**) LVDD, (**C**) LVESD.

15 articles involving 1,404 patients reported the LVEF outcome. The LVEF of patients who engaged in TCE was significantly improved compared with the control group based on a random-effects model (MD = 4.58, 95%CI: 3.28–5.88, *P* < 0.00001, *I*^2 ^= 79%) (Figure 3A).

Eight studies involving 786 patients reported the LVDD outcome. The LVDD of patients who engaged in TCE was significantly less compared with the control group based on a random-effects model (MD = −3.83, 95%CI: −5.27–−2.38, *P* < 0.00001, *I*^2 ^= 94%) (Figure 3B).

Four studies involving 456 patients reported the LVESD outcome. The LVESD of patients who engaged in TCE was significantly less compared with the control group based on a random-effects model (MD = −2.17, 95%CI: −4.10–−0.24, *P* < 0.00001, *I*^2 ^= 96%) (Figure 3C).

#### 3.3.3. Effects of TCE on physical function

**Figure 4:**
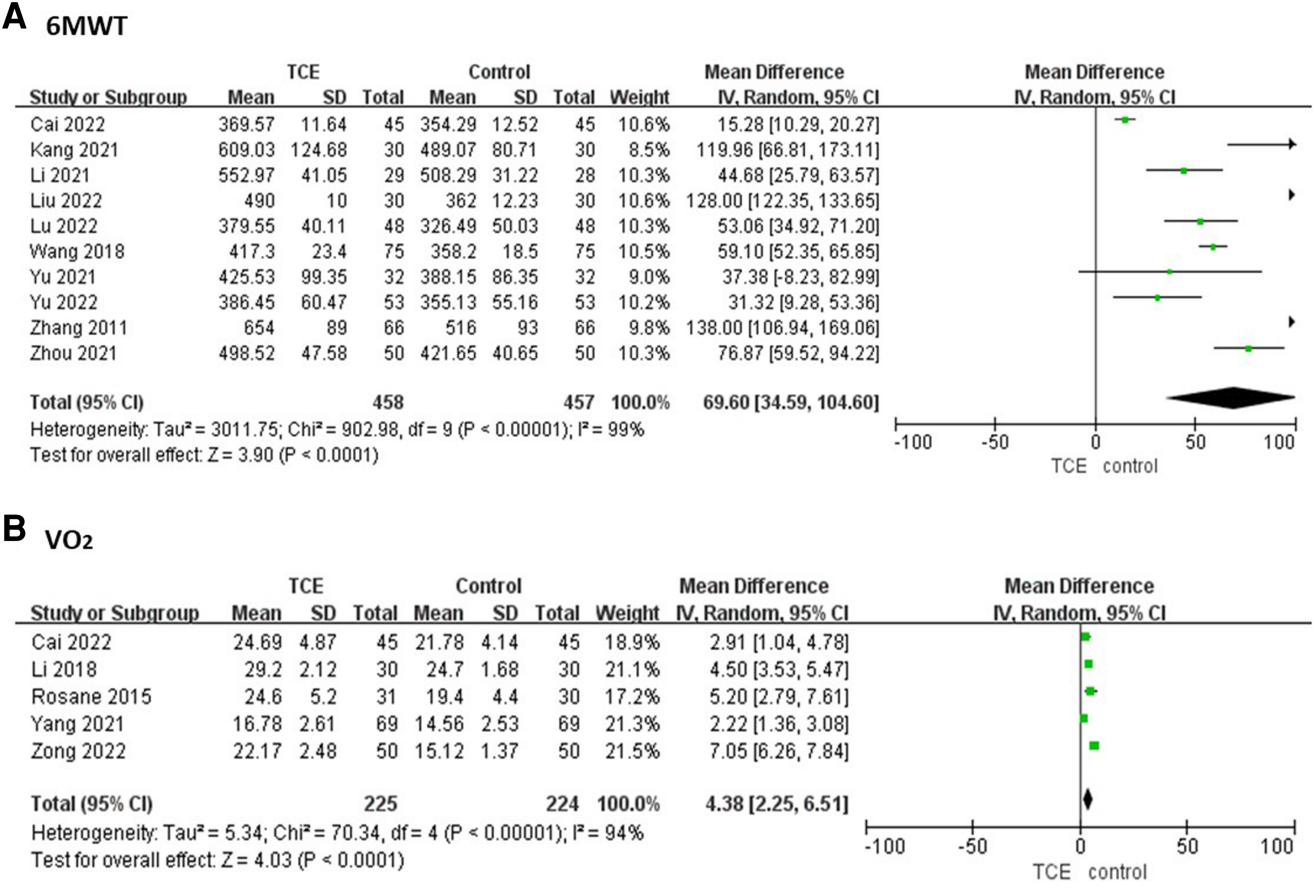
The forest plots of (**A**) 6MWT, (**B**) VO_2._

Ten studies involving 915 patients reported the 6MWT outcome. The 6MWT of patients who engaged in TCE was significantly improved compared with the control group based on a random-effects model (MD = 69.60, 95%CI: 34.59–104.60, *P* < 0.00001, *I*^2 ^= 99%) (Figure 4A).

Five studies involving 448 patients reported the VO_2_ outcome. The VO_2_ of patients who engaged in TCE was significantly improved compared with the control group based on a random-effects model (MD = 4.38, 95%CI: 2.25–6.51, *P* < 0.00001, *I*^2 ^= 94%) (Figure 4B).

#### 3.3.4. Effects of TCE on quality of life

**Figure 5:**
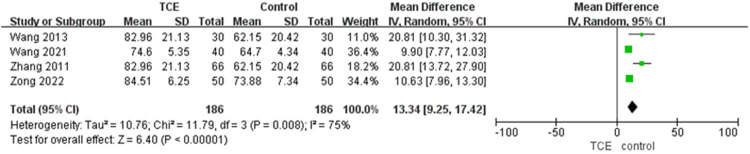
The forest plot of SF-36.

Four studies involving 372 patients estimated the quality of life with SF-36 outcome. The SF-36 score of patients who engaged in TCE was significantly improved compared with the control group based on a random-effects model (MD = 13.34, 95%CI: 9.25–17.42, *P* = 0.008, *I*^2 ^= 75%) (Figure 5).

#### 3.3.5. Effects of TCE on emotional distress

**Figure 6:**
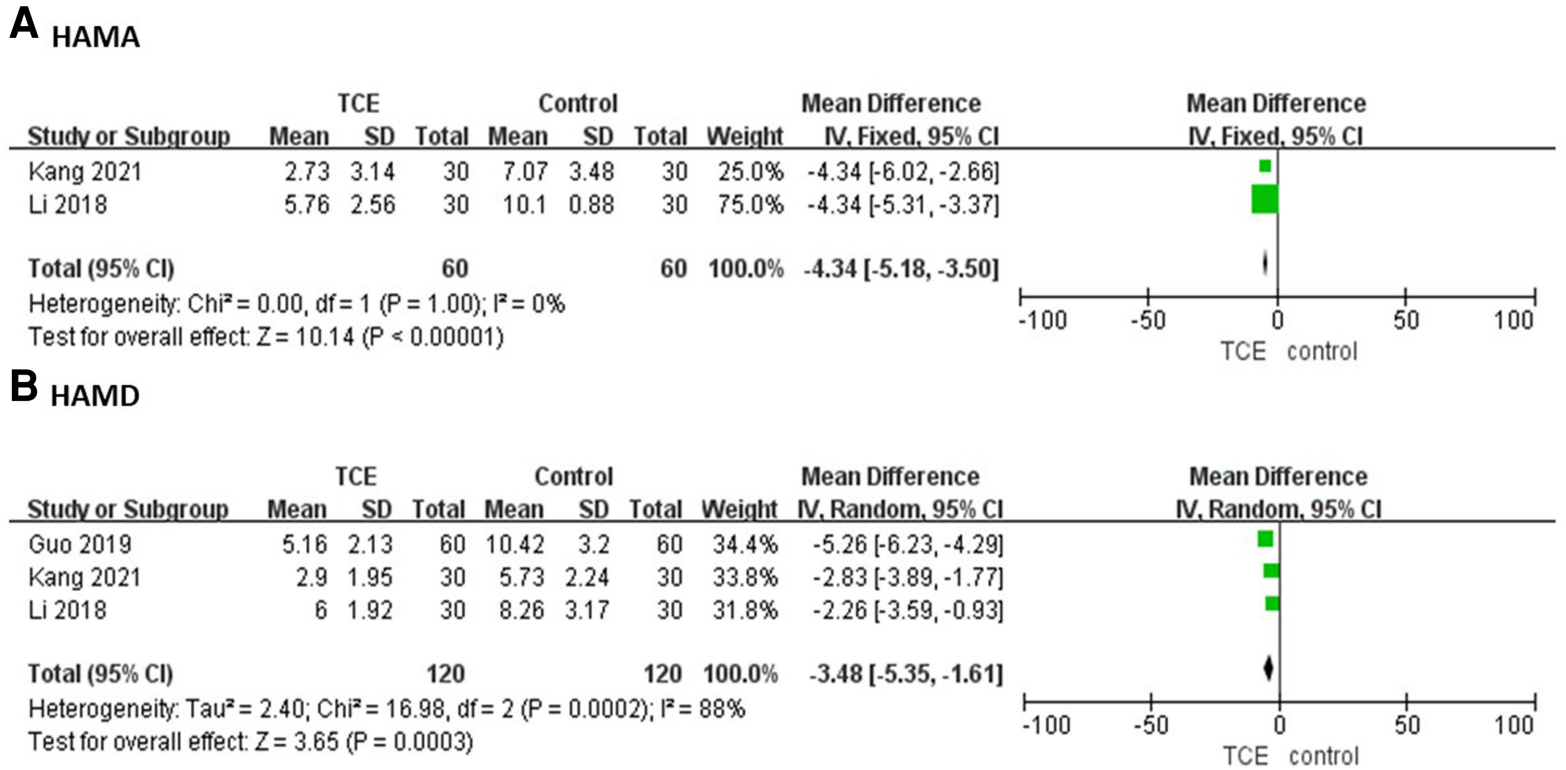
The forest plots of (**A**) HAMA, (**B**) HAMD.

Two studies involving 120 patients reported the HAMA outcome. The HAMA of patients who engaged in TCE was significantly less compared with the control group based on a fixed-effects model (MD = −4.34, 95%CI: −5.18–−3.50, *P* = 1.00, *I*^2 ^= 0%) (Figure 6A).

Three studies involving 240 patients reported the HAMD outcome. The HAMD of patients who engaged in TCE was significantly less compared with the control group based on a random-effects model (MD = −3.48, 95%CI: −5.35–−1.61, *P* = 0.0002, *I*^2 ^= 88%) (Figure 6B).

#### 3.3.6. Effects of TCE on MCAEs

**Figure 7:**
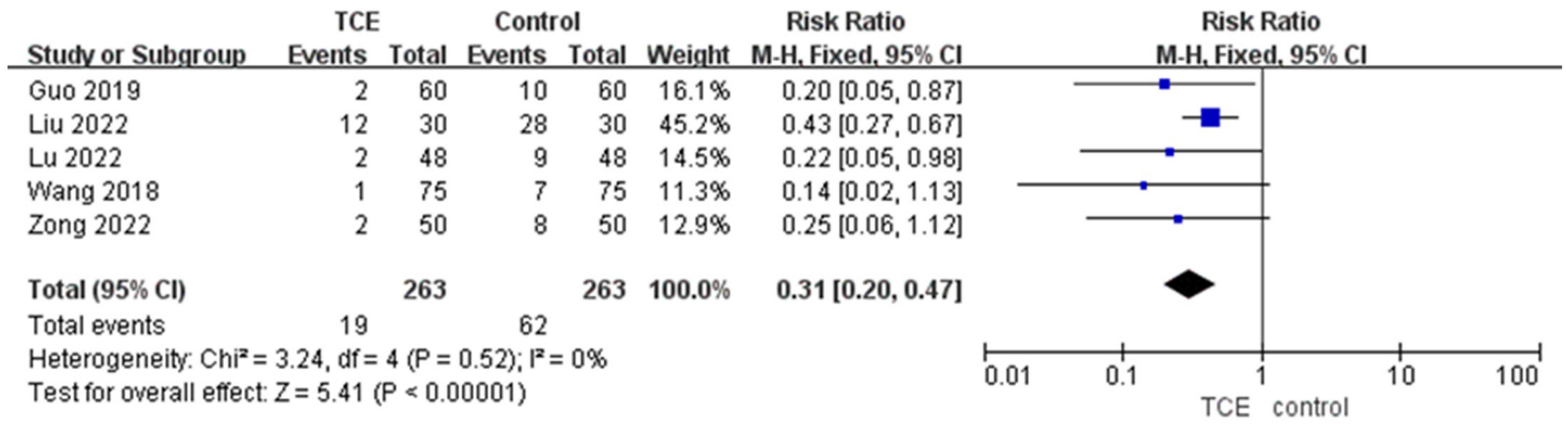
The forest plot of MCAEs.

Five articles involving 526 patients reported the MACEs outcome. The incidence of MACEs of patients who engaged in TCE was significantly less compared with the control group based on a fixed-effects model (RR = 0.31, 95%CI: 0.20–0.47, *P* = 0.52, *I*^2 ^= 0%) (Figure 7).

### 3.4. Sensitivity analysis and subgroup analysis

Sensitivity analyses were performed by excluding each study one by one. The results revealed NT-proBNP, LVEF, LVDD, LVESD, 6MWT, and VO_2_ outcomes were not significantly altered. The sensitivity of the SF-36 outcome decreased when Zhang's study (24) was removed (*I*^2^ changed to 50%), and the sensitivity of the HAMD outcome decreased when Guo's study (20) was excluded (*I*^2^ changed to 0%).

Meanwhile, we implemented subgroup analyses of NT-proBNP, LVEF, 6MWT, VO_2_, and SF-36 outcomes according to the type of TCE in Table 2. There are three subgroups depending on the type of exercise: Tai Chi, Ba Duan Jin, and Tai Chi combined Ba Duan Jin. As shown the heterogeneity of subgroup analyses in NT-proBNP, LVEF and SF-36 outcomes significantly decreased.

**Table 2:** Subgroup analyses.

Outcome	Subgroup	Number of studies	Sample size	MD (95%CI)	Statistical method	*P* value for heterogeneity	*I* ^2^
NT-proBNP	Ba Duan Jin	4	310	−67.85 [−107.84, −27.87]	Random-effects	<0.00001	89%
	Tai Chi	3	204	−147.93 [−344.66, 48.81]	Random-effects	<0.00001	99%
LVEF	Ba Duan Jin	10	972	3.93 [3.25, 4.61]	Fixed-effects	0.10	39%
	Tai Chi	3	276	6.45 [0.25, 12.65]	Random-effects	<0.00001	95%
	Ba Duan Jin and Tai Chi	2	156	6.23 [4.30, 8.16]	Fixed-effects	0.18	44%
6MWT	Ba Duan Jin	6	602	54.57 [29.03, 80.11]	Random-effects	<0.00001	97%
	Tai Chi	3	253	73.86 [9.35, 138.36]	Random-effects	<0.00001	93%
VO_2_	Ba Duan Jin	2	190	5.07 [1.02, 9.21]	Random-effects	<0.00001	94%
	Tai Chi	2	199	3.49 [0.60,6.38]	Random-effects	0.02	81%
SF-36	Ba Duan Jin	2	180	10.18 [8.52, 11.85]	Fixed-effects	0.68	0%
	Tai Chi	2	192	20.81 [14.93, 26.69]	Fixed-effects	1.0	0%

## 4. Publication bias

**Figure 8:**
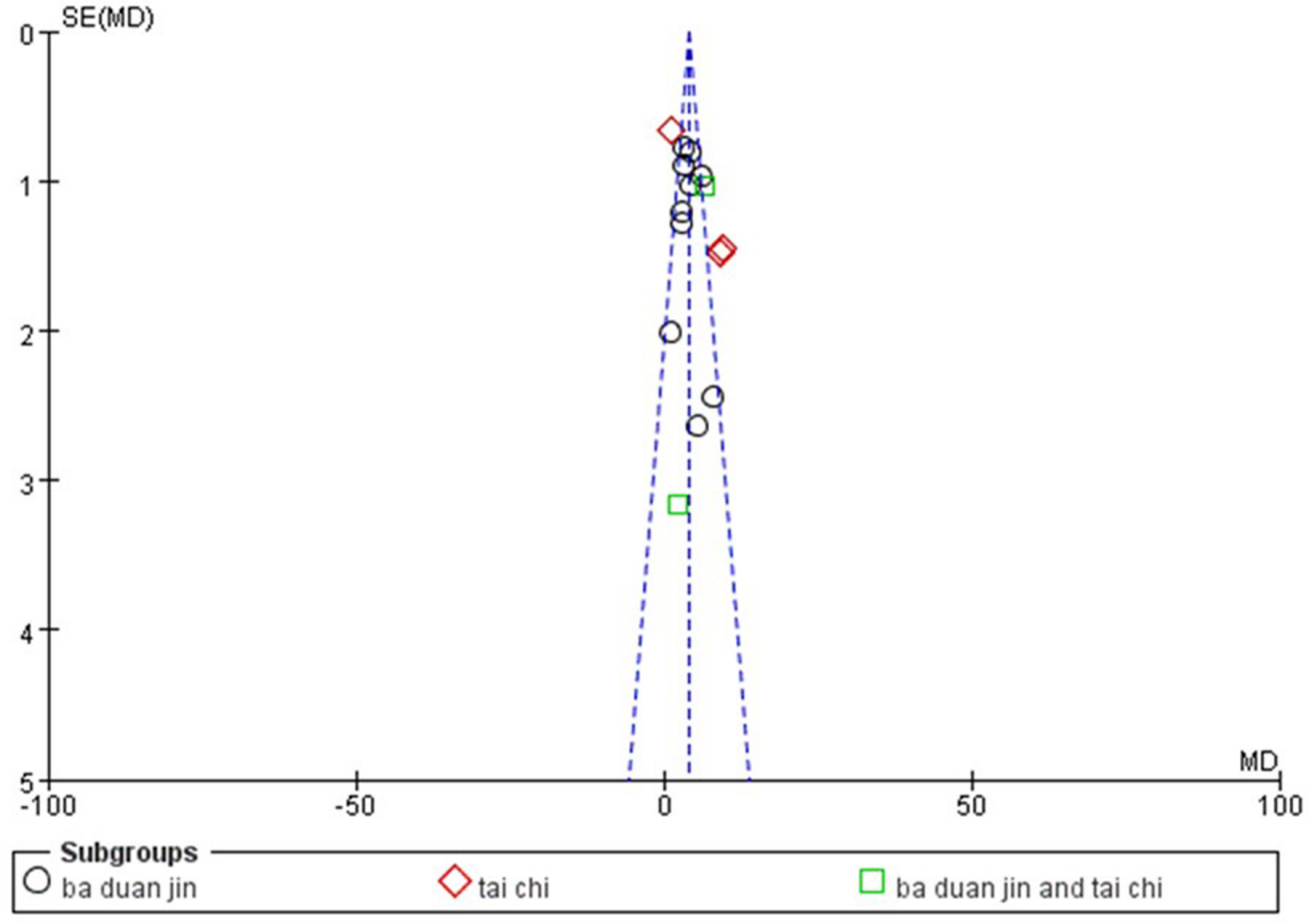
Funnel plot of LVEF.

Funnel plots were used to assess the publication bias of the 21 included articles. We drew a funnel plot with the LVEF outcome divided into three subgroups as an example. As seen in Figure 8, all studies were relatively evenly distributed and located on both sides of the axis, and the bias was acceptable.

## 5. Discussion

MI is a high-incidence disease worldwide, bringing a heavy social burden, especially for low and middle-income countries (33). Numerous studies have indicated that PCI only solves the blockage problem of blood vessels, but long-term rehabilitation exercises are still needed to restore myocardial function (34, 35). The common belief among patients is that after a MI, exercise should be avoided. However, patients who have undergone PCI or CABG should participate in a clinically supervised exercise program. According to Fernandez's study (36), patients who engage in CR have a 26% reduction in mortality and an 18% reduction in hospital readmission rates compared to patients who do not participate in CR. Exercises for post-MI rehabilitation are a crucial part of enhancing patients' quality of well-being and long-term prognosis following drug and surgical treatment. Additionally, it plays a key role in cardiac remodeling and cardiovascular regulation (37). Although CR is strongly recommended as a post-MI treatment, the current underuse is concerning (38, 39). Thus, our meta-analysis focused on MI patients. In recent years, TCEs such as Tai Chi, Qi Gong, and Ba Duan Jin have become increasingly popular around the world because of their gentle movements, low-risk, easy training, ancd long-term adherence. TCE is not only effective in regulating the physical condition and mental health of patients with Parkinson's disease, type 2 diabetes, chronic obstructive pulmonary disease, chronic pain disorders, and cancer but also beneficial in improving the cardiopulmonary function and quality of life (7).

A total of 21 RCTs comprising 1,890 patients were included for the first time to perform the meta-analysis. Unfortunately, Tai Chi and Ba Duan Jin were the only TCEs included. In comparison to the control group, patients in the TCE group demonstrated better results in NT-proBNP, LVEF, LVDD, LVESD, 6MWT, VO_2_, SF-36 scores, HAMA, and HAMD scale outcomes, and showed a decrease in the MACEs outcome. In addition, Ba Duan Jin and Tai Chi were likewise discovered to be safe, with no instances of TCE-related adverse events. This is consistent with Ting Liu's meta-analysis, Tai Chi increases social well-being and physical endurance in CHD patients (40). A randomized controlled trial reported that adapted personalized motor activity could improve health in individuals with mental disorders and physical comorbidities (41). Notably, our study further demonstrated that TCE improved the emotions of patients with MI as well. About 50% of patients experience anxiety after MI (42). Due to diminished cardiopulmonary function, which affects their capacity for employment and social contact as well as their long-term drug dependence, frequent hospitalizations, and significant financial stress, they are more prone to anxiety, depression, and other negative emotions. Therefore, TCE is very suitable and effective for MI patients to improve their mental and physical function. Apart from that, some researchers believed that TCE might influence cholesterol (18) and myocardial fibrosis levels (23) throughout the screening. However, the number of studies was not enough to perform a meta-analysis.

Furthermore, we conducted sensitivity analysis and subgroup analysis to investigate the source of heterogeneity. Except for HAMA and MACEs outcomes, some heterogeneity in the results of other outcomes may be due to certain clinical and methodological variations in different trials. Subgroup analysis revealed differences in TCE types as a potential source of heterogeneity and suggested that Tai Chi might be more efficient than Ba Duan Jin.

TCE involves the purposeful regulation of breath and thought, in harmony with the control of the body. Combining self-awareness with physical posture, the flow of breath, and stillness of thought is considered an exercise that activates the natural self-regulatory capacity to stimulate a balanced release of endogenous neurohormones (43). Overall, our meta-analysis provided reliable results in terms of TCE improving CR in patients with MI.

### 5.1. Limitations

This study has several limitations. Firstly, there is a lack of gray literature. If the findings from unpublished studies differ from the published data, the meta-analysis may become biased. Secondly, most of the included literature did not explicitly mention allocation concealment and only one study was blinded, so selection bias may be present. Finally, only Ba Duan Jin and Tai Chi were discussed in this essay. The majority of the included RCTs were Chinese, so language restrictions might lead to publication bias.

## 6. Conclusion

In conclusion, the use of TCE in MI patients showed significant improvements in cardiac function, physical function, quality of life, and mental health, also reduced the incidence of MACEs, which has good clinical application value and is recommended as a complementary therapy for CR in MI patients. It was found that Tai Chi might be more efficient than Ba Duan Jin. However, the above findings still need to be verified by more high-quality RCTs. The specific frequency and duration of different exercises should be analyzed to prescribe the most appropriate exercise prescription in the future.

## Data Availability

Publicly available datasets were analyzed in this study. The data analyzed in this article can be found here: PubMed (https://pubmed.ncbi.nlm.nih.gov/). Web Of Science (https://clarivate.com/products/web-of-science/). Cochrane library (https://www.cochranelibrary.com/). Embase (https://www.embase.com/). CNKI (https://www.cnki.net/). VIP (http://Lib.cqvip.com/). Wan Fang Databases (https://www.wanfangdata.com.cn/index.html). SinoMed (http://www.sinomed.ac.cn/index.jsp).
