# Supplementary material for: Effects of traditional Chinese exercises on cardiac rehabilitation in patients with myocardial infarction: a meta-analysis of randomized controlled trials
Source: Front Cardiovasc Med. 2023 Oct 2;10:1223677. doi: 10.3389/fcvm.2023.1223677 (PMC10577298; doi:10.3389/fcvm.2023.1223677)
Supplement: Supplementary file 1 [file Table1.docx]

Supplementary Material

Effects of traditional Chinese exercises on cardiac rehabilitation in patients with myocardial infarction： A meta-analysis of randomized controlled trials

Jingfang Zhang^1^, Jieqiong Weng^1^, Mengfei Yuan^1^, Xingchen Shen^1^, Yayi Weng^1^, Xiaoxu Shen^1*^

*** Correspondence:** Xiaoxu Shen, MD, Ph. D, Cardiology Department, Dongzhimen Hospital, Beijing University of Chinese Medicine, Beijing 100700, China. Email: shenxiaoxupaper@sina.com

# Supplementary Tables

Supplementary Table 1 Summary of the quality assessment by The Cochrane Collaboration's tool of included studies

| Study | Random  sequence  generation | Allocation  concealment | Blinding of  participants  and personnel | Binding of  outcome  assessment | Incomplete  outcome data | Selective  reporting | Other bias |
| --- | --- | --- | --- | --- | --- | --- | --- |
| Wang2018^12^ | Low | Unclear | Unclear | Unclear | Low | Low | Low |
| Cai2022^13^ | Low | Unclear | Unclear | Unclear | Low | Low | Low |
| Wang2021^14^ | Low | Unclear | Unclear | Unclear | Low | Low | Low |
| Zhang2019^15^ | Low | Unclear | Unclear | Unclear | Low | Low | Low |
| Zhou2021^16^ | Low | Unclear | Unclear | Unclear | Low | Low | Low |
| Zong2022^17^ | Low | Unclear | Unclear | Unclear | Low | Low | Low |
| Kang2021^18^ | Low | Unclear | Unclear | Unclear | Low | Low | Low |
| Liu2022^19^ | Low | Unclear | Unclear | Unclear | Low | Low | Low |
| Guo2019^20^ | Low | Unclear | Unclear | Unclear | Low | Low | Low |
| Li2018^21^ | Low | Unclear | Unclear | Unclear | Low | Low | Low |
| Yang2021^22^ | Low | Unclear | Unclear | Unclear | Low | Low | Low |
| Yu2021^23^ | Low | Unclear | Unclear | Unclear | Low | Low | Low |
| Zhang2011^24^ | Unclear | Unclear | Unclear | Unclear | Low | Low | Low |
| Wang2013^25^ | Unclear | Unclear | Unclear | Unclear | Low | Low | Low |
| Liu2017^26^ | Unclear | Unclear | Unclear | Unclear | Low | Low | Low |
| Lu2022^27^ | Low | Unclear | Unclear | Unclear | Low | Low | Low |
| Yu2022^28^ | Low | Unclear | Unclear | Unclear | Low | Low | Low |
| Li2021^29^ | Low | Unclear | Unclear | Unclear | Low | Low | Low |
| Mao2020^30^ | Low | Unclear | Unclear | Unclear | Low | Low | Low |
| Chen2020^31^ | Low | Unclear | Unclear | Unclear | High | Low | Low |
| Rosane2015^32^ | Low | Unclear | Unclear | Low | Low | Low | Low |
